# Alcohol Avoidance Training as a Mobile App for Problem Drinkers: Longitudinal Feasibility Study

**DOI:** 10.2196/16217

**Published:** 2020-04-14

**Authors:** Melissa C Laurens, Marcel E Pieterse, Marjolein Brusse-Keizer, Elske Salemink, Somaya Ben Allouch, Ernst T Bohlmeijer, Marloes G Postel

**Affiliations:** 1 Centre for eHealth and Well-being Research University of Twente Enschede Netherlands; 2 Research Group Technology, Health & Care Saxion University of Applied Sciences Enschede Netherlands; 3 Medical School Twente Medisch Spectrum Twente Enschede Netherlands; 4 Department of Psychology University of Amsterdam Amsterdam Netherlands; 5 Department of Clinical Psychology University of Utrecht Utrecht Netherlands; 6 Digital Life Amsterdam University of Applied Sciences Amsterdam Netherlands; 7 Tactus Addiction Treatment Enschede Netherlands

**Keywords:** mobile alcohol avoidance training, approach bias, cognitive bias modification, alcohol

## Abstract

**Background:**

Alcohol use is associated with an automatic tendency to approach alcohol, and the retraining of this tendency (cognitive bias modification [CBM]) shows therapeutic promise in clinical settings. To improve access to training and to enhance participant engagement, a mobile version of alcohol avoidance training was developed.

**Objective:**

The aims of this pilot study were to assess (1) adherence to a mobile health (mHealth) app; (2) changes in weekly alcohol use from before to after training; and (3) user experience with regard to the mHealth app.

**Methods:**

A self-selected nonclinical sample of 1082 participants, who were experiencing problems associated with alcohol, signed up to use the alcohol avoidance training app Breindebaas for 3 weeks with at least two training sessions per week. In each training session, 100 pictures (50 of alcoholic beverages and 50 of nonalcoholic beverages) were presented consecutively in a random order at the center of a touchscreen. Alcoholic beverages were swiped upward (away from the body), whereas nonalcoholic beverages were swiped downward (toward the body). During approach responses, the picture size increased to mimic an approach movement, and conversely, during avoidance responses, the picture size decreased to mimic avoidance. At baseline, we assessed sociodemographic characteristics, alcohol consumption, alcohol-related problems, use of other substances, self-efficacy, and craving. After 3 weeks, 37.89% (410/1082) of the participants (posttest responders) completed an online questionnaire evaluating adherence, alcohol consumption, and user satisfaction. Three months later, 19.03% (206/1082) of the participants (follow-up responders) filled in a follow-up questionnaire examining adherence and alcohol consumption.

**Results:**

The 410 posttest responders were older, were more commonly female, and had a higher education as compared with posttest dropouts. Among those who completed the study, 79.0% (324/410) were considered adherent as they completed four or more sessions, whereas 58.0% (238/410) performed the advised six or more training sessions. The study identified a significant reduction in alcohol consumption of 7.8 units per week after 3 weeks (95% CI 6.2-9.4, *P*<.001; n=410) and another reduction of 6.2 units at 3 months for follow-up responders (95% CI 3.7-8.7, *P*<.001; n=206). Posttest responders provided positive feedback regarding the fast-working, simple, and user-friendly design of the app. Almost half of the posttest responders reported gaining more control over their alcohol use. The repetitious and nonpersonalized nature of the intervention was suggested as a point for improvement.

**Conclusions:**

This is one of the first studies to employ alcohol avoidance training in a mobile app for problem drinkers. Preliminary findings suggest that a mobile CBM app fulfils a need for problem drinkers and may contribute to a reduction in alcohol use. Replicating these findings in a controlled study is warranted.

## Introduction

Problematic alcohol use is one of the most prevalent health problems in modern life. It has several negative personal, social, and economic consequences [1-4]. When not addressed properly and timely, problematic alcohol use can result in alcohol use disorder (AUD). Regular treatment of AUD and support for reducing problematic alcohol use, such as cognitive behavioral therapy and motivational interviewing, primarily focus on influencing controlled cognitive mechanisms. Although these treatments have proven to be effective [5,6], long-term outcomes remain modest [7]. To achieve progress in the effectiveness of treatments, research should further investigate the role played by relatively automatic processes. The dual process model [8,9] integrates both relatively slow reflective processes and fast impulsive processes.

Cognitive bias modification (CBM) programs have been developed to influence these impulsive processes by, for example, changing biases in action tendencies [10]. Research demonstrates that problem drinkers have an approach bias for alcohol-related stimuli [11]. Different CBM programs have been developed to directly influence the approach bias, for example, the stimulus response compatibility task [11] where participants are required to make a symbolic approach/avoidance movement to pictures and alcohol avoidance training, which is an adapted version of the alcohol approach avoidance task (A-AAT) [12]. In alcohol avoidance training, participants respond to either alcoholic or nonalcoholic pictures of beverages on a screen by pulling toward or pushing away the pictures using a joystick or keyboard. An important feature of alcohol avoidance training is the zooming function, which follows the pushing or pulling movement, creating the sensation of the beverage moving either away or toward the user. The use of alcohol avoidance training has shown positive results in a clinical setting [13], where receiving four sessions of alcohol avoidance training displayed a long-term clinical effect in alcohol-dependent patients (n=214) when added to their regular treatment. This study and a large replication study (n=509) [14] illustrated significant reductions in relapse a year after treatment (13%, *P*=.05 and 10%, *P*=.04, respectively) in the CBM condition as compared with a placebo condition. This effect was found to be mediated by a change in approach tendencies in the latter study [15]. Additionally, a recent study comparing different combinations of approach bias and attention bias retraining to “sham” or no training with 1405 alcohol-dependent patients obtained a somewhat smaller but significant result (*P*=.04), showing on average a 8.4% higher success rate 1 year after treatment; however, it did not confirm the mediating effect of the change in approach tendencies on the outcome [16].

Nowadays, most bias modification training programs are offered in a laboratory setting, clinical setting, or online via a computer. Although transferring treatment from a face-to-face setting to a mobile setting could be accompanied by lower patient engagement and higher dropout rates [17], online training programs have the advantage that participants can use the intervention independent of time and place [18], thus making it particularly suitable for outpatient treatment. For example, Wiers et al [19] conducted a Web-based CBM study on self-selected problem drinkers (n=136). Participants in the different conditions (including the control condition) of the study reduced their alcohol intake by 2.31 to 9.94 units per week [19]. However, having to log onto a computer or laptop for every training session may hinder motivation to train [20]. As most adults use a smartphone or tablet daily [21] and other forms of CBM training are operated by a joystick or keyboard, offering CBM training on a mobile device is an intuitive next step. Delivering CBM training this way facilitates more frequent training, as it allows participants to perform each session anywhere and anytime and may therefore promote engagement. A small study by Boendermaker et al [22] found support for this assumption, as participants (young and regular drinkers, not specifically selected on the basis of their motivation to reduce their drinking behavior) appeared to be more involved with CBM training when using a smartphone version of CBM training than when training on a computer. Until now, however, little is known about the use and evaluation of mobile CBM training in people who are willing to change their drinking behavior.

In the present study, a smartphone/tablet version of alcohol avoidance training was tested among a self-selected sample of Dutch problem drinkers from the general population. The aims of this study were to (1) measure adherence to mobile alcohol avoidance training; (2) determine the change in weekly alcohol use from before to after training; and (3) assess user experience.

## Methods

### Design

This pilot study consisted of a single group design with the following three measurements: baseline measurement, postintervention assessment at 3 weeks, and follow-up assessment at 3 months. The study was approved by the ethics committee within the faculty of Behavioral Management & Social Sciences of the University of Twente (approval number: BCE16395).

### Participants

Participants were recruited between November 10 and November 23, 2016, via free publicity in national and regional newspapers and on radio stations and television. A total of 1214 participants signed up for the study. To be included, participants had to (1) be willing to reduce/stop their drinking habit or be concerned about their drinking habit; (2) be aged 18 years or older; (3) have access to and ability to use the internet via a smartphone or tablet; (4) have the ability to read and write in Dutch; and (5) provide (online) informed consent.

### Intervention

**Figure 1:**
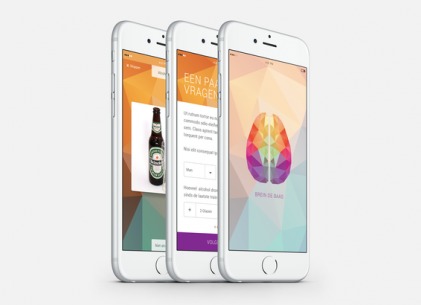
Example of the Breindebaas app.

The Breindebaas app (Figure 1) is a mobile version of alcohol avoidance training [13,14], which is an adapted version of the AAT [23]. The mobile version distinguishes itself from the original (joystick operated) and online (keyboard operated) versions of alcohol avoidance training by (1) using swiping movements on the screen (directly touching the picture and swiping it away with a finger) and (2) asking the participant to react to the actual content of the picture (relevant feature) instead of the orientation of the picture (irrelevant feature). Every session contained 100 pictures (drinks only, without context) from the Amsterdam Beverage Picture Set [24], and of these, half depicted alcoholic beverages and the other half depicted nonalcoholic beverages. Participants were instructed to respond to these pictures by swiping the alcoholic beverages away from them and swiping the nonalcoholic beverages toward them. Participants were encouraged to swipe as quickly and accurately as possible. If a mistake was made, such as reacting too slowly and not completing the “swipe movement” correctly, participants received a short error notification (text and sound) with instructions. When swiping correctly, a sound notified participants of their correct response. After every 20, 50, and 80 pictures, participants received an encouraging message on the screen of their device, such as “you’re well on your way” or “almost there!.” These messages were included to motivate participants to complete their training session. Between every two pictures, there was a 1-second interval. The time interval between an encouraging message and the next picture was 2 seconds. After every session, the participants received an overview of their score regarding their average time and percentage of correct responses.

### Measures

Table 1 presents an overview of the characteristics and measures assessed at baseline, postassessment, and follow-up. Internal consistency of scales was assessed with Cronbach alpha, with values ≥.7 being considered as acceptable [25].

**Table 1:** Characteristics and measures at baseline, postassessment, and follow-up.

	Baseline	Postassessment	Follow-up
Sociodemographics	√		
TLFB^a^	√	√	√
AUDIT^b^	√		
Other substances	√		
OCDS^c^	√		
DRSEQ^d^	√		
CSQ^e^		√	
User friendliness		√	
Treatment history		√	√
Use of the app + reasons		√	√

^a^TLFB: time-line follow-back.

^b^AUDIT: Alcohol Use Identification Test.

^c^OCDS: Obsessive Compulsive Drinking Scale.

^d^DRSEQ: Drinking Refusal Self-Efficacy Questionnaire.

^e^CSQ: Client Satisfaction Questionnaire.

### Sociodemographic Characteristics

Participants reported their gender, birth date, source of income, daily occupation, educational level, and smartphone/tablet information (type, brand, and model).

### Alcohol Consumption

The Dutch adaptation [26] of the self-reported time-line follow-back (TLFB) procedure [27] was used to assess alcohol consumption. Participants indicated the number of standard units of alcohol consumed throughout each day over the past week. The total score of the scale was calculated by the total sum of all 7 days [27]. TLFB is a highly used retrospective estimation measure, and it has been used with similar target groups [28], with adequate validity [29].

### Alcohol-Related Problems

The 10-item Dutch version [30] of the Alcohol Use Disorders Identification Test (AUDIT) [31] was used to assess drinking behaviors and alcohol-related problems. Internal consistency in the current sample was acceptable with a Cronbach alpha of .8.

### Use of Other Substances

Participants were asked about their prior use over the past year and current use of other substances (tobacco, cannabis, cocaine, lysergic acid diethylamide, amphetamines, XTC, GHB, opiates, benzodiazepines, and others).

### Drinking Refusal Self-Efficacy

Items of the Drinking Refusal Self-Efficacy Questionnaire (DRSEQ) [32] were used to assess the following three dimensions of self-efficacy in relation to refusal of alcohol, as identified by Young et al [33]: social pressure, emotional relief, and opportunistic drinking. The original DRSEQ contains a total of 31 items for these three dimensions, and it has shown good psychometric quality, both for the subscales and for the total scale (Cronbach alpha >.8) [34]. For this study, a short measure of self-efficacy was constructed using three items from both the dimensions of social pressure and emotional relief and two items from the dimension of opportunistic drinking, representing the items that were most relevant for our study. Cronbach alpha for these eight items was .9 in the current sample.

### Craving

Using the five-item scale [35] of the original 14-item Obsessive Compulsive Drinking Scale [36], participants were asked to rate their thoughts, feelings, and actions concerning alcohol. Cronbach alpha in the current sample was .74.

### Adherence

Adherence was measured by recoding the self-reported number of sessions. Participants were advised to complete at least six training sessions; these were established as the mean optimum in a study by Eberl et al [37]. In our study, completing four or more of the advised six training sessions was considered as adherent, as research by Wiers et al previously showed this to have a significant effect (*P*=.05) [13].

### User Satisfaction

User satisfaction regarding the CBM alcohol avoidance training was assessed using the Dutch version of the eight-item Client Satisfaction Questionnaire (CSQ) [38]. Cronbach alpha was .91.

### User Experience

Participants were invited to answer several questions about their experience with the Breindebaas app. Questions concerned its overall impression, benefits and drawbacks in using the app, suggestions for future development, main reason behind using the app, technical problems, use of other alcohol intervention treatments during the intervention period, place of using the app (eg, at home, at work, and in the pub), and intention to continue using the app in the future. Lastly, participants were questioned on concentration, which was measured by simply inquiring about their general concentration during a training session, on a 4-point scale (not concentrated at all to very much concentrated), which was converted to a dichotomous variable (1-2: not concentrated; 3-4: concentrated) for analyses. At follow-up, participants were asked whether they had kept on using the app and their reasoning behind this decision. They were also asked whether they used other forms of help associated with their alcohol use during the research period.

### Procedure

Participants were referred to a website [39], where information about the study and the app was provided. Participants who demonstrated an interest were then asked to fill out a digital informed consent form and an online baseline questionnaire. Upon completion, instructions for downloading the app and an access code needed for using the app were provided digitally. Participants were requested to complete at least two training sessions every week for 3 weeks, leaving at least 24 hours between two sessions. Three days after completing a session, participants received an alert that a new training session was available. If participants did not finish a training session within 5 days, a reminder was sent via push message. Optionally, participants could choose to receive these messages via short message service text messaging. The link to the postassessment questionnaire was sent via email 3 weeks after the start of the training. Participants were not given specific instructions to keep on using the app after the postassessment. Three months after completing the postassessment questionnaire, participants were asked to fill out a follow-up questionnaire. A reminder was sent by email or short message service text messaging 1 week later. By completing all three questionnaires, participants had a chance of winning one of five available gift vouchers, each worth 100 euros.

### Statistical Analysis

Descriptive statistics were used to describe the baseline characteristics of the participants and the characteristics of those who completed the training at posttest. Means and standard deviations (SDs) or medians and interquartile ranges (IQRs) are provided depending on the normality distribution for continuous variables. Categorical variables are presented as numbers with corresponding percentages. Independent samples *t*-tests or Wilcoxon rank-sum tests (continuous variables) and chi-square tests or Fisher exact tests (categorical variables) were used to compare baseline characteristics between posttest responders and posttest dropouts, as well as between follow-up responders and follow-up dropouts. A paired samples *t*-test was performed to compare alcohol consumption at baseline and posttest. Linear regression analysis was performed to identify any predictors correlated with a change in alcohol consumption between baseline and posttest. Variables associated (*P*≤.15) in univariate analysis were all entered in the multivariate model, and subsequently, they were eliminated step by step based on significance (backward elimination method).

Changes in alcohol consumption from baseline to postassessment at 3 weeks and to follow-up assessment at 3 months were analyzed using a mixed-model analysis. In case of significant changes over time, Sidak post-hoc analyses were performed to test which measurements were statistically significantly different. All tests were performed using SPSS version 24.0 (IBM Corp, Armonk, New York).

## Results

### Baseline Characteristics

In total, 1238 participants completed the baseline questionnaire. Of these, 156 participants were excluded owing to age <18 years (n=2), not signing the informed consent form (n=22), duplicate records (n=3), or having a nonalcohol-related reason to participate (n=129), eg, professional interest in the app. Thus, 1082 participants were included for analysis at baseline. Table 2 demonstrates the baseline characteristics of the 1082 participants. The sample contained slightly more male participants (58.4%), with an overall mean age of 49.9 (SD 11.3) years. The mean weekly alcohol consumption was 36.6 (SD 24.5) standard units. Among the participants, 93.53% (1012/1082) reported an AUDIT score ≥8, indicating problematic alcohol use throughout the vast majority of the sample.

### Posttest Responders and Adherence

Among the original 1082 participants, 410 (37.89%) completed the postintervention assessment (referred to as posttest responders), with 206 participants (19.0%) also completing the follow-up assessment after 3 months (referred to as follow-up responders). Posttest responders (n=410) and posttest dropouts (n=672) were compared regarding baseline characteristics (Table 2). Posttest responders were significantly older (*P*<.001), were less often male (*P*=.01), had a higher education (*P*<.001), and consumed less alcohol (*P*<.001) as compared with posttest dropouts. This was mainly caused by lower consumption of alcohol by females in the completer group. Furthermore, posttest responders had lower AUDIT scores among both males and females and lower DRSEQ scores mainly among males. Finally, posttest responders used fewer other substances.

**Table 2:** Baseline characteristics and differences in baseline characteristics between posttest responders and posttest dropouts.

Variable		Total (n=1082)	Posttest responders (n=410)	Posttest dropouts (n=672)	Analysis *t* value	χ^2^ *P* value
Age (years), mean (SD)		49.89 (11.32)	52.4 (10.2)	48.3 (11.7)	5.80	<.001^a^
**Gender, n (%)**					6.14	.01^a^
	Male	632 (58.4)	220 (53.7)	412 (61.3)	—^h^	—
	Female	450 (41.6)	190 (46.3)	260 (38.7)	—	—
Employed, n (%)		726 (70.9)	271 (70.6)	455 (71.1)	0.03	.86
**Education, n (%)**					26.60	<.001^a^
	High^b^	583 (57.0)	257 (66.8)	326 (51.1)	—	—
	Middle^c^	286 (28.0)	91 (23.6)	195 (30.6)	—	—
	Low^d^	154 (15.1)	37 (9.6)	117 (18.3)	—	—
**Weekly alcohol consumption, mean (SD)**		36.6 (24.5)	33.3 (21.8)	38.7 (25.8)	−3.69	<.001^a^
	Male	42.4 (26.5)	40.0 (24.7)	43.7 (27.3)	−1.71	.09
	Female	28.5 (18.7)	25.5 (14.4)	30.7 (21.0)	−3.08	<.001^a^
**AUDIT** ^e^ **, mean (SD)**		17.2 (6.7)	15.8 (6.1)	18.0 (6.9)	−5.27	<.001^a^
	Male	18.2 (6.3)	17.1 (6.0)	18.8 (6.5)	−3.13	<.001^a^
	Female	15.7 (6.8)	14.4 (5.9)	16.7 (7.3)	−3.71	<.001^a^
**DRSEQ** ^f^ **, mean (SD)**		25.4 (7.4)	24.8 (7.4)	25.8 (7.3)	−2.17	.03^a^
	Male	25.0 (7.3)	24.1 (7.3)	25.4 (7.3)	−2.21	.03^a^
	Female	26.0 (7.4)	25.6 (7.5)	26.4 (7.3)	−1.06	.29
OCDS^g^, mean (SD)		5.3 (3.2)	5.2 (3.0)	5.4 (3.3)	−1.31	.19
**Other substances, n (%)**		452 (41.8)	141 (34.4)	311 (46.3)	14.8	<.001^a^
	Tobacco	338 (31.2)	100 (24.4)	238 (35.4)	14.41	<.001^a^
	Benzodiazepines	142 (13.1)	45 (11.0)	97 (14.4)	2.67	.10
	Cannabis	108 (10.0)	24 (5.9)	84 (12.5)	12.52	<.001^a^
	Others	209 (19.3)	—	—	—	—

^a^*P*<.05 (two-tailed).

^b^University of research or university of professional education.

^c^Higher general secondary education or intermediate vocational education.

^d^Primary school or lower vocational education.

^e^AUDIT: Alcohol Use Identification Test.

^f^DRSEQ: Drinking Refusal Self-Efficacy Questionnaire.

^g^OCDS: Obsessive Compulsive Drinking Scale.

^h^Not applicable.

In a similar analysis, among 410 participants remaining in the study at posttest, baseline characteristics were compared between follow-up responders (n=206) and follow-up dropouts (n=204). A significant difference in baseline characteristics was found regarding participant age, with follow-up responders tending to be older (mean 55.2 vs 52.5 years, *P*=.01), and their use of tobacco, with follow-up responders smoking significantly less (30.6% [63/206] vs 38.2% [78/204], *P*=.01). No other significant differences were found.

Participants reported completing from 1 to over 10 sessions in the questionnaire (median 6, IQR 4-7). Of the 410 posttest responders, 323 (78.8%) completed four or more sessions, which was considered adherent in this study. Furthermore, 239 (58.3%) participants performed the recommended six sessions (n=123, 30.0%) or more (n=116, 28.4%). The main reasons for not completing the recommended number of sessions were “it does not seem to help me” (21.0%, n=86) and “not having enough time” (19.0%, n=78).

Concentration while performing a session was recoded as a dichotomous variable (concentrated/not concentrated). Of the 410 posttest responders, 375 (91.5%) reported to be concentrated throughout their training sessions.

### Changes in Alcohol Consumption Over Time and Predictors

The average alcohol consumption of the posttest responders (n=410) decreased significantly by 7.8 units per week (95% CI 6.2-9.4, *P*<.001) from baseline (mean 33.3 [SD 21.8]) to postassessment (mean 25.5 [SD 20.4]).

Table 3 illustrates the results of the regression analyses, which evaluated the potential predictors of changes in alcohol use. The following variables were found to be univariately associated with a stronger decrease in alcohol consumption (*P<.*15): male gender, unemployment, high level of baseline craving, high baseline AUDIT score, high level of self-reported concentration during sessions, and high adherence. When these factors were entered in a multivariate regression model, only gender, adherence, and craving remained significant predictors of a change in alcohol consumption.

### Changes in Alcohol Use at Follow-up

The subsample of 206 participants that completed the follow-up assessment displayed a reduction in alcohol use over time. Their mean weekly alcohol consumption decreased from 31.6 (SD 23.2) units at baseline to 24.4 (SD 19.2) units at 3 weeks and to 18.2 (SD 17.3) units at follow-up 3 months later, resulting in a total decrease of 13.4 units a week. Pairwise comparisons in mixed-model analysis demonstrated the reductions for this subsample as significant both from baseline to postassessment (mean difference 7.2, CI 4.9-9.6, *P*<.001) and from postassessment to follow-up (mean difference 6.2, CI 3.7-8.7, *P*<.001).

A mixed model for repeated measurements, in which all 410 participants were taken into account, produced similar results (Table 4). Pairwise comparisons in mixed-model analysis also displayed the reductions as significant both from baseline to postassessment (mean difference 7.4, CI 5.7-9.6, *P*<.001) and from postassessment to follow-up (mean difference 6.6, CI 4.2-9.0, *P*<.001).

**Table 3:** Univariate and multivariate linear regression coefficients, confidence intervals, and significance levels of baseline characteristics with regard to alcohol consumption.

		Univariate coefficient	95% CI	*P* value	Multivariate coefficient	95% CI	*P* value
**Gender**							
	Male	9.92	0.75-7.09	.02	4.44	1.36-7.52	.01
	Female	Reference			Reference		
**Concentration**					N/A	N/A	N/A
	No	Reference					
	Yes	6.74	1.08-12.40	.02			
**Adherence**							
	No	Reference			Reference		
	Yes	5.69	1.83-9.55	<.001	6.09	2.33-9.85	<.001
**Work situation**					N/A	N/A	N/A
	Unknown	Reference					
	Paid	1.54	−2.06 to 5.14	.04			
	Unpaid	6.67	0.07-13.27	.01			
OCDS^a^		1.15	0.63-1.68	<.001	1.15	0.64-1.67	<.001
AUDIT^b^		0.05	0.23-0.75	<.001	N/A	N/A	N/A

^a^OCDS: Obsessive Compulsive Drinking Scale.

^b^AUDIT: Alcohol Use Identification Test.

**Table 4:** Mean alcohol consumption at baseline, postassessment, and follow-up using mixed models.

	Mean alcohol consumption (SD)	
Measurement	Subsample of participants who completed the follow-up assessment (n=206)	Total participants (n=410)
Baseline	31.6 (1.6)	32.6 (0.9)
Postassessment	24.4 (1.3)	25.2 (0.9)
Follow-up	18.2 (1.2)	18.6 (1.0)

### User Experiences

When posttest responders (n=410) were asked about what they gained from using the app over a 3-week period, almost half of the participants stated having the feeling of more control over their drinking (eg, gained more control over alcohol use, decided more frequently not to drink, and chose to drink alcohol less automatically)*,* with many participants also reporting being more aware of their alcohol use (36.1%, 148/410). However, 47.3% (194/410) of participants reported that they gained nothing from using the app.

Posttest responders had an overall CSQ score of 20.9 (SD 4.4) with an average score of 2.6 on a scale from 1-4 (item variances: 0.5), indicating moderate satisfaction. Participants were particularly positive about the simple, fast-working, user-friendly design of the app. Criticism and subsequent suggestions about the app mostly targeted elements concerning monotony and lack of personalization. Participants deemed app use as boring and monotonous owing to the repetition of the task and pictures. They suggested introducing motivational elements, such as levels or game options, as well as a shorter interval between swiping movements and subsequent pictures. Thus, the introduction of a greater variation in pictures and the possibility of choosing pictures was suggested.

Of the 410 posttest responders, 318 (77.6%) had never sought help or used an intervention to reduce alcohol use previously. Additionally, 46 (11.2%) participants reported receiving extra help in reducing alcohol use during the Breindebaas training period in the form of a self-help program, help from a general practitioner, help from a professional in (addiction) care, or peer support. Of these 46 participants, 20 had never sought help before.

Of the 206 participants that completed the follow-up questionnaire, 85 continued to use the app. The main reasons behind this decision were that using the app helped them to be more conscious of their alcohol use (n=51) and it assisted in maintaining their reduced drinking habit (n=15). Of the 121 participants who stopped using the app, the main reasons were doubts regarding the app’s effectiveness (n=40) and simply forgetting to use the app (n=33).

## Discussion

To our knowledge, this study is the first to evaluate a mobile version of AAT training in a sample of problem drinkers among the general population. Given the debate on the effectiveness of CBM [40,41], it is essential to differentiate between experimental studies with students, which are set up to show that biases can be influenced (but do not always show a change in behavior), clinical trials with alcohol dependent patients who are motivated to change [42], and studies with nonclinical problem drinkers from the general public. Gaining more insight into the feasibility and outcomes of CBM for the participants in this study, who were not clinically diagnosed with AUD but were willing to change their drinking behavior, is therefore especially relevant.

The baseline characteristics, adherence to the intervention, change in alcohol consumption, and user experiences were studied. Participants in this study were comparable to groups analyzed in previous research via Web-based self-help interventions regarding the level of problematic alcohol use [42-44] and no active search for professional help to aid the reduction of their drinking behavior [45,46]. We were pleasantly surprised by the large group of problem drinkers interested in using the Breindebaas app, considering the short timeframe of the study. It can be considered a strength that this low-threshold application seems to appeal to this hard-to-reach group, as it may reduce the stigma associated with directly meeting a professional [47].

The intervention adherence among posttest responders was high. Most of the posttest responders (78.8%, 323/410) used the app four or more times, doing better than an online CBM trial by Wiers et al on alcohol, where 43.3% (136/314) of the participants completed the prescribed four sessions [19]. The fact that the Breindebaas app is a mobile version of an AAT and therefore available to participants at any moment could be a particular contributing factor. For example, a pilot study using a mobile CBM app on obesity found a training session completion rate of 86% (17/20) [48].

In this study, we observed a significant reduction in alcohol use among posttest responders immediately after using the mobile intervention and 3 months later. Although a reduction of 13 units per week is substantial for such a brief intervention, the results need to be seen in the context of a pilot study without a control condition for comparing the findings, especially given earlier observed main effects across CBM and control conditions [19]. Further research should be implemented, in which the app training should be compared with sham training in a controlled design. The same caution should be exercised with the impact of the predictors (gender, adherence, and craving) that were established for changing an individual’s alcohol consumption.

Participants were mostly positive about the Breindebaas app. The simple, fast-working, user-friendly design resulted in participants reporting more awareness and control over their alcohol use. Nevertheless, a considerable portion of participants also reported gaining nothing from using the app. Reportedly, this was because of the repetitious and monotonous characteristics of the AAT and its lack of personalization. Regarding the lack of personalization, participants mainly indicated that some of the pictures contained beverages (both alcoholic and nonalcoholic) that were not appealing to them at all. Wiers et al already indicated that personalizing alcohol-related stimuli is a potential way forward [41]. Studies on CBM related to eating habits indicated that personalizing CBM tasks may increase attention, motivation, and interest and therefore may increase adherence [49,50]. An additional reason for withdrawing from using the Breindebaas app was the participant’s questions and doubts pertaining to the working mechanism behind the training. Other studies support this finding [20], which may mean that for the future development of similar tasks, explaining the reasoning and the importance of repeated training is crucial.

A number of limitations should be addressed. First, as this study was set up as a pilot study with the aim to assess feasibility and adherence, no control group was allocated. Consequently, the change in self-reported alcohol consumption found in this study may well be the result of a placebo effect of the app, a nonspecific effect of engaging in any intervention, or even an effect of participating in a study. As already mentioned, just reporting alcohol use alone can have an effect on the reduction of drinking [51]. Nonetheless, the change in alcohol consumption demonstrated by participants in this study seems large enough to justify future studies on the effectiveness of the Breindebaas app. Second, participants were invited to partake in this study via free publicity channels and were only asked to provide basic information about themselves. As none of the participants of this study had face-to-face interactions, we needed to rely on their self-reporting. This, of course, could decrease the reliability of our results, although the reliability of the measures was described as acceptable to good in this study. Self-reported alcohol use reduction among subjects participating in treatment is likely to be positively biased, overestimating outcomes. In addition to self-reporting, the fact that more than half of the participants who had originally signed up dropped out during the training period and only 19% completed the follow-up questionnaire may have decreased the generalizability of the results. The dropout attrition rate in this study was comparable to that in other online CBM studies on alcohol or smoking [19,52,53]. Given the design of the study, the most likely factors influencing the dropout rate (between baseline and follow-up) were (1) ease of enrolment; (2) ease of dropout; (3) no personal contact via face-to-face interviews or telephone; and (4) fully paid for intervention [54]. Finally, no approach bias measurements were made before and after using the app. Therefore, it is unknown whether the approach bias of participants changed over time or whether this mediated the effect of training on alcohol use. Previous studies have indicated that relevant treatment effects of CBM on clinical outcomes almost always are accompanied by a decrease in cognitive bias [55,56]. A study by Eberl et al showed that patients with a strong approach bias at baseline elicited the best results in decreasing their bias [14], whereas no overall approach bias was established in the sample. This might be associated with the ambivalent stance that many patients with AUD hold with respect to alcohol [57]. Future developments in mobile CBM applications and research should therefore consider incorporating bias measurements, providing more insight into the working mechanism of CBM in the subclinical population.

In summary, several suggestions from users and researchers provide the following insights for the future development of the Breindebaas app: (1) using personalized stimuli in the app; (2) adding more information about the working mechanism and effects of CBM, which can increase motivation; (3) including bias measurement in the app, so participant progress in bias scores can be tracked; and (4) adding motivational/gamification elements (eg, levels and rewards) to improve user adherence to the app. The addition of gamification elements was mentioned by users of the Breindebaas app and has shown promising results in other forms of cognitive training [22,58]. In addition to app development, more research on the effects of using the Breindebaas app in controlled trials is advised. One suggestion is a three-armed study, in which participants are assigned to either training with the Breindebaas app, a mobile intervention with self-monitoring and goal setting features, or a waiting-list condition. As the Breindebaas app contains relevant feature approach/avoidance training, developing a credible placebo version is very challenging. Using a different mobile intervention to rule out nonspecific effects seems like a pragmatic choice. Following up on this research, approaching the same target group (problem drinkers from the general population) would be consistent. This is the first study in which alcohol avoidance training was adapted to a mobile app for problem drinkers. User evaluation suggests that this CBM app fulfils a need for problem drinkers reluctant to seek clinical treatment, as the majority of the sample never sought help prior to the study and had been mostly positive about using the app. Participants in this study reduced their alcohol intake by a total of 13 units per week. Although the results should be interpreted cautiously owing to the absence of a control group, adoption of the CBM app may contribute to reducing alcohol use among those who experience problems associated with drinking.

## Abbreviations

AAT: approach avoidance task
AUD: alcohol use disorder
AUDIT: Alcohol Use Disorders Identification Test
CBM: cognitive bias modification
CSQ: Client Satisfaction Questionnaire
DRSEQ: Drinking Refusal Self-Efficacy Questionnaire
IQR: interquartile range
SD: standard deviation
TLFB: time-line follow-back
